# The Potential of Members of the Apple Sub‐Family Maloideae Against Obesity and Metabolic Disorders: A Review

**DOI:** 10.1002/fsn3.70934

**Published:** 2025-09-18

**Authors:** Zainab R. Abdelrahman, Mai S. Maaliah, Shtaywy S. Abdalla

**Affiliations:** ^1^ Department of Biological Sciences, School of Science The University of Jordan Amman Jordan

**Keywords:** *Crataegus*, *Cydonia*, *Eriobotriya*, maloideae, *Malus*, metabolic disorder, obesity

## Abstract

Metabolic disorder, and obesity in particular, is a global epidemic among the world's population and is a complex multifactorial health problem. Obesity is associated with serious health risks like diabetes, coronary heart disease, non‐alcoholic fatty liver disease, hyperlipidemia, and hyperglycemia disorders and has shown a steady increase in morbimortality indicators. Many drugs have been approved for effective treatment of metabolic disorders and their symptoms, but the cost on the health system and on the individual patient is extremely high. Therefore, an adjunctive treatment for managing obesity and metabolic disorder could be in the use of medicinal plants and functional foods, which could reduce the cost as well as reduce the side effects of these medications. Traditional herbal medicines and functional foods have become the subject of global importance, with both medical and economic implications. The apple subfamily Maloideae includes commercially and medicinally valuable fruits like apples, pears, loquat, quince, and hawthorn, and many other plants. This subfamily has a distinctive fruit, the pome, with characteristic antioxidant content. This review summarizes the recently published research, preclinical data, brief phytochemistry, and pharmacology on 5 Maloideae genera to underscore their potential as adjunctive therapy against obesity and metabolic disorder and future research opportunities.

## Graphical abstract

The apple subfamily Maloideae includes commercially and medicinally valuable plants. The fruits and other plant parts contain antioxidant compounds, like polyphenols. When consumed, the fruit and other plant parts mostly reduce body weight in humans. Studies showed that fruits and other plant parts have an impact against obesity and metabolic disorder.

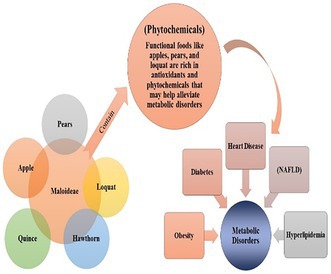

## 1 Introduction

Obesity is known as an excessive and abnormal accumulation of fat in different body organs (Chen et al. 2024; Chandrasekaran and Weiskirchen 2024). It has also been defined as extra weight associated with adverse health consequences (Bessesen 2008). Many researchers consider obesity a chronic disease (Melson et al. 2024; Busetto et al. 2024; Nagpal et al. 2024). Obesity is classified as one of the components of metabolic syndrome, which is characterized by high blood triglycerides, increased low‐density lipoproteins, and reduced high‐density lipoproteins, high blood pressure, impaired fasting glucose, and insulin resistance (Maaliah et al. 2024).

Many factors play significant roles in obesity prevalence including biological, behavioral, socio‐economic factors, and type of gender (Muscogiuri et al. 2024). Other factors that may increase the risk of obesity include nutrition, exposure to air pollutants, smoking habits as well as chemical substances which somehow enter the human body and cause an increase in adipocytes (Nogueira‐de‐Almeida et al. 2024). Nutritional habits, like consumption of processed food and low intake of the Mediterranean diet, are associated with obesity leading to food addiction or “binge eating disorder” (D'Innocenzo et al. 2019) which is a pathological case where obese people spend a lot of time thinking about food; they can't moderate their food intake and therefore consume a large quantity of food in a short time (Rossi et al. 2024; Lucas et al. 2022). Poor lifestyle and low physical activity influence the progress of obesity and this can be worsening by the advanced technology in social media, including using mobile phone, watching TV and working on computers (Lucas et al. 2022).

Many studies highlighted the worldwide prevalence of obesity. For example, during 2020–2023, the highest prevalence of overweight and obesity was in Polynesia (New Zealand and other islands in the central and south Pacific Ocean) and the lowest was in middle and Western Africa (Zhang et al. 2024). The prevalence of obesity in the Middle East between 2000 and 2020 significantly increased from 14.5% to 40.6% of the population and the highest obesity was found in Kuwait and Syria while the lowest obesity was found in Yemen (Okati‐Aliabad et al. 2022). In the USA, it was found that one out of five children and adolescents were obese in 2017–2018 and similar prevalence was observed in the UK (Tsoi et al. 2022). During August 2021 to August 2023, the prevalence of obesity among adults in the USA increased to 40.3% (Emmerich et al. 2024).

Many diseases are associated with obesity, such as diabetes type 2, hypertension, hyperlipidemia, obstructive sleep apnea, and myocardial infarction (Chen et al. 2024). Additionally, sarcopenic obesity, a combination of sarcopenia and obesity, increases the cardiovascular risk and mortality (Atkins and Wannamathee 2020). Moreover, ectopic obesity, the deposition of fat around organs, leads to insulin resistance, fatty liver, inflammation, cardiac steatosis, and coronary heart disease (Britton and Fox 2011) and obese people, in general, are more likely to have depression (Zhang et al. 2024).

Due to the complications and rapid prevalence of obesity and costly anti‐obesity drugs, it was necessary to find natural, safe treatments with fewer side effects and affordable adjunctive treatments with notable results (Hidalgo‐Lozada et al. 2024). These natural treatments do not necessarily form substitutes for the approved obesity medications but rather form adjunct therapy that reduces the cost and reduces the side effects of the approved medications. Numerous publications studied the effect of many plants and herbs on obesity and metabolic syndrome. Similarly, many plant products have been used as alternative medicines to treat obesity and its complications (Maaliah et al. 2024; Abdelrahman et al. 2023).

The Maloideae subfamily (the apple subfamily) members are cultivated mostly for their fruits, besides other reasons. Their fruits contain many active ingredients that showed many therapeutic effects (Robertson et al. 1992; Rohrer et al. 1994). Among these ingredients are the flavonoids, the phenolic acids, and the essential oils, all of which are endowed with antioxidant potential as well as a wide spectrum of pharmacological effects (Section 2.1). In the current review, we will highlight the role of five members of Maloideae as anti‐obesity natural products. Many species of this subfamily are abundantly consumed as foods all around the globe, and their anti‐obesity effects cannot be ignored.

## 2 Research Status on Maloideae Genera and Their Potential in Alleviating Obesity and Metabolic Disorder

**FIGURE 1:**
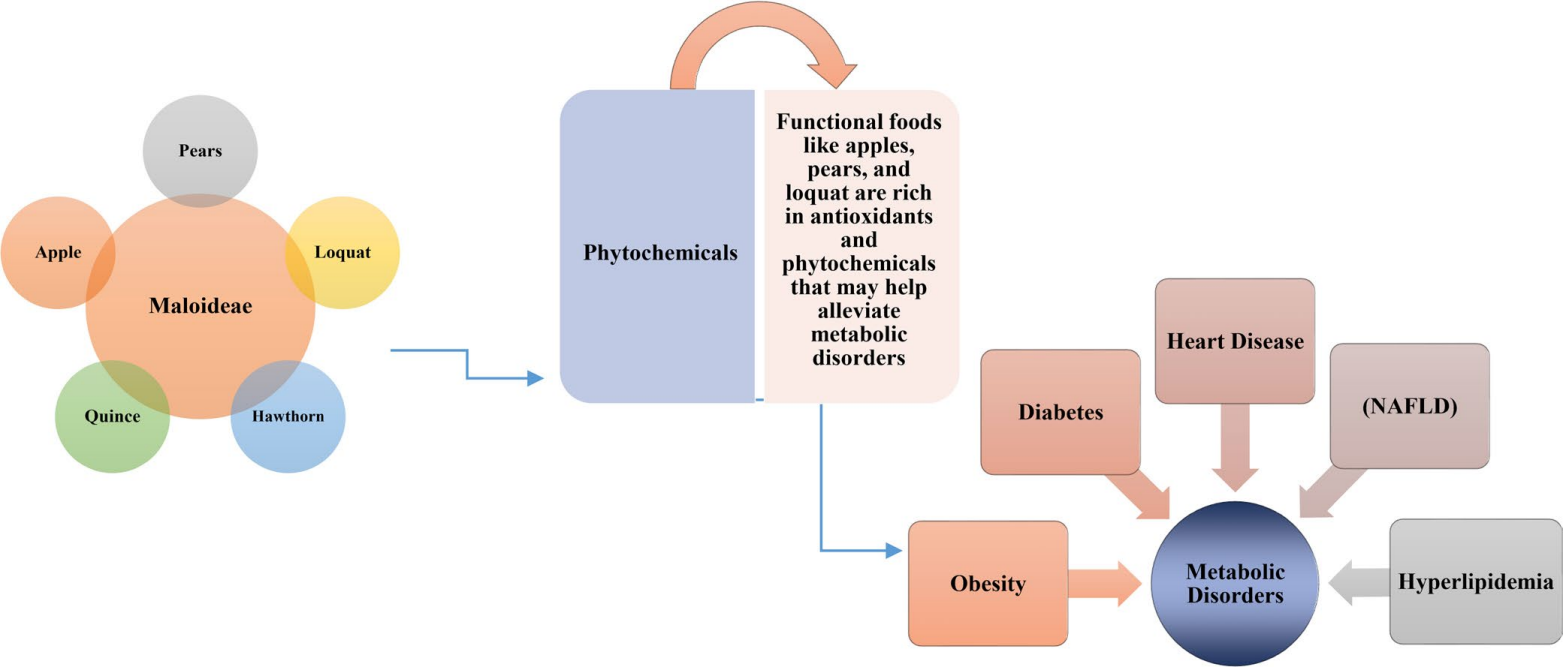
The Maloideae subfamily includes plants like apple, pear, loquat, hawthorns, and quince. These plants produce phytochemicals like flavonoids, triterpenes, and antioxidants that have known effects against metabolic disorders like obesity, diabetes, cardiovascular disease, non‐alcoholic fatty liver disease (NAFLD), and hyperlipidemia.

Rosaceae is a plant family consisting of about 100 genera with 3000 species (Pathak et al. 2019). These are categorized into four subfamilies: Rosoideae, Spiraeoideae, Maloideae, and Amygdaloideae (Pathak et al. 2019; Dickinson and Campbell 1991). Maloideae is the largest subfamily of Rosaceae, with around 28 genera and approximately 940 species (Robertson et al. 1992). The familiar Maloideae genera are *Malus* (apples), *Pyrus* (pears), *Eriobotrya* (loquat), *Cydonia* (quince) and *Crataegus* (hawthorn) (Robertson et al. 1992; Evans and Dickinson 2005). Maloideae species are cultivated for their edible fruits (Figure 1) and as an ornament, and they are mainly grown throughout the northern hemisphere (Rohrer et al. 1994).

### 2.1 Phytochemistry of Maloideae

The phytochemistry of the individual species of Maloideae has been the subject of intense investigation, and many publications have been produced. These investigations are summarized in Table 1. The major classes of the chemical compounds isolated from the apple subfamily include:

- Flavonoids, like quercetin, rutin, morin, myricetin, kaempferol, naringin, apigenin, hesperidin, epicatechin, isorhmnetin, dihydrochalcones, and anthocyanins, are generally having a wide spectrum of activity but specifically are of paramount significance in alleviating metabolic disorders.
- Phenolic acids, like benzoic, hydroxybenzoic, gallic, vanillic, coumaric, ferulic, ursolic, malic, caffeic, quinic, lactic, succinic, sorbic, protocatechuic, and salicylic acids, are usually having significant antioxidant effects that endow them with biological activity in lipid metabolism.
- Essential oils that include saturated and polyunsaturated fatty acids such as linoleic, oleic, and palmitic acids
- Vitamins including vitamin A, niacin, riboflavin, thiamin, B12, folate, and vitamins C, D, and E.
- Minerals including Ca^2+^, Fe, Mg, P, K, Zn, and Cu.

**TABLE 1:** Phytochemistry of 5 Maloideae genera.

Genus/Common name	Chemical constituents
*Malus* (Apples)	From the fruit of *M. hupehensis*, *M. domestica* , *M. pumila* and many other species: Polyphenols (hydroxybenzoic acid, gallic acid, protocatechuic acid; hydroxycinnamic acids in conjugated form such as quinic acid and caffeic acid) (Bator et al. 2024; Asma et al. 2023) Flavonoids (flavonols, flavanols‐3‐ols, anthocyanins, and dihydrochalcones) (Bator et al. 2024) Vitamins include (vitamin A, niacin, riboflavin, thiamin, vitamin B12, vitamin D, vitamin C, and folate) (Bator et al. 2024; Shirosaki et al. 2012) Minerals (calcium, iron, magnesium, phosphorus, potassium, sodium and zinc) (Bator et al. 2024) From the seeds of *M. pumila* : amygdalin, a cyanogenic glycoside, dihydrochalcones, β‐sitosterol, stigmasterol, campesterol and vitamin E (Shirosaki et al. 2012) From the leaves of *M. pumila* : Phloridzin (Shirosaki et al. 2012) From the peels of *M. domestica* : Rich in phenols, ascorbic acid, glutathione, hydroxycinnamic acids, flavonols, and anthocyanins than flesh (Popiolek‐Kalisz et al. 2024)
*Pyrus* (Pears)	From the fruits of *P. communis* , *P. ussuriensis* *and* *P. pyrifolia* : Vitamin C, vitamin A, vitamin E and vitamin B3, B5, B12, carotenoids, and anthocyanins (Nazir et al. 2020) Phenolic acids include arbutin, gallic, chlorogenic, caffeic, vanillic, coumaric, dehydroascorbic, and ferulic acids, arbutin, ursolic acid, kaempferol (Nazir et al. 2020; Hong et al. 2021) Flavonoids such as quercetin and isorhamnetin, epicatechin and proanthocyanidins (Nazir et al. 2020; Hong et al. 2021) Triterpenes (oleanolic acid and ursolic acid, carotenoids and anthocyanins) (Hong et al. 2021) Asian pear is richer in phenolic compounds with less sugar than European pear (Hong et al. 2021) Sugars includes (catechol, erythritol, and L‐arabinitol) (Boby et al. 2022) From the leaves of *P. ussuriensis* : Arbutin, isoquercitrin, sorbitol, ursolic acid, kaempferol glucopyranoside, quercetin glucopyranoside (Peng et al. 2022) From the bark of *P. ussuriensis* : Α‐glucosidase, friedelin, epifriedelanol, and β‐sitosterol, and the root contains phloridzin (Peng et al. 2022) From the flowers of *P. communis* and *P. ussuriensis* : Chlorogenic acid, fatty acid and pectin (Peng et al. 2022; Velmurugan and Bhargava 2013) From the peels of *P. pyrifolia* and *P. ussuriensis* : Phenolic acids, flavonoids and triterpenes (Wang et al. 2015; Mahdy et al. 2023)
*Eriobotrya* (loquat)	From the fruits of *E. japonica* : Glycosides, terpenoids such as sesquiterpenes (Abdelrahman et al. 2023) Flavonoids such as epicatechin, epicatechin gallate, methyl chlorogenate, cinchonain and quercetin (Abdelrahman et al. 2023) Phenolic compounds including hydroxycinnamic acid derivatives such as chlorogenic acid, neochlorogenic acid, methyl‐chlorogenic acid, feruloylquinic acid, caffeoylquinic acid, hydroxybenzoic acid, ferulic acid, and oleanolic acid, ursolic acid, hydroxyursolic acid, gallic acid, caffeic acid, ellagic acid, and catechin (Abdelrahman et al. 2023) From the leaves of *E. japonica* : Triterpenoid, flavonoid, essential oil, tannins, and megastigmane glycoside (Shih et al. 2010; Li, Li, et al. 2020) From the seeds of *E. japonica* Flavonoids, ellagic acid, tannins, and amygdalin (Abdelrahman et al. 2023; Tanaka et al. 2008)
*Crataegus* (Hawthorn)	From the fruits of *C. monogyna* : Vitamins such as B1, B2, B6, and C, and 17 amino acids (Radi et al. 2023) Phenolic acids such as gallic acid, chlorogenic acid, ferulic acid, caffeic acid and rosmarinic acid, ascorbic acid, coumaric acid, citric acid and catechols (Radi et al. 2023) Flavonoids such as rutin and catechin, tannins such as tannic acid (Radi et al. 2023) Rutin and chlorogenic acid could be used as indicators for the classification of *Crataegus* species for example, *C. pinnatifida* and *C. cuneata* had chlorogenic acid but not rutin in their fruit. *C. dachurica* and *C. maximowiczii* did not contain either chlorogenic acid or rutin, *C. shensiensis* contained rutin but not chlorogenic acid (Guo and Jiao 1995) From the leaves of *C. laevigata* : Apigenin glucoside Apigenin glucoside, oligomeric procyanidins, epicatechin and flavonoid (Guo and Jiao 1995; Svedström et al. 2002)
*Cydonia* (Quince)	From the fruits of *C. oblonga* : Organic acids, free amino acids, polyphenolic compounds such as malic acid, mandelic acid, caffeic acid, quercetin, catechin hydrate and morin (Aslam and Hussain 2013; Mirmohammadlu et al. 2015) From the pulp and peel of *C. oblonga* Caffeoylquinic acid, rutin, and kaempferol 3‐rutinoside (Mirmohammadlu et al. 2015)

In addition, many species contain essential amino acids, terpenoids, and tannins.

### 2.2 *Malus* (Apple)

*Malus* is a common tree that grows naturally but is mostly cultivated globally. Apples are commonly eaten; more than 59 million tons were consumed around the world in 2019; they form a significant part of the human diet as well as processed food products including juices, jams, pies, vinegar, wines, and chips (Asma et al. 2023; Silva et al. 2002; Wolfe et al. 2003). Fuji, Pumila, Cortland, Golden Delicious, Rome Beauty, Idared, Red Delicious, Granny Smith, and Gola are major apple species (Shirosaki et al. 2012; Wolfe et al. 2003). Species differ in their anthocyanin content, which gives apples a reddish color (Wolfe et al. 2003).

#### 2.2.1 Effect on Obesity and Metabolic Disorder

**FIGURE 2:**
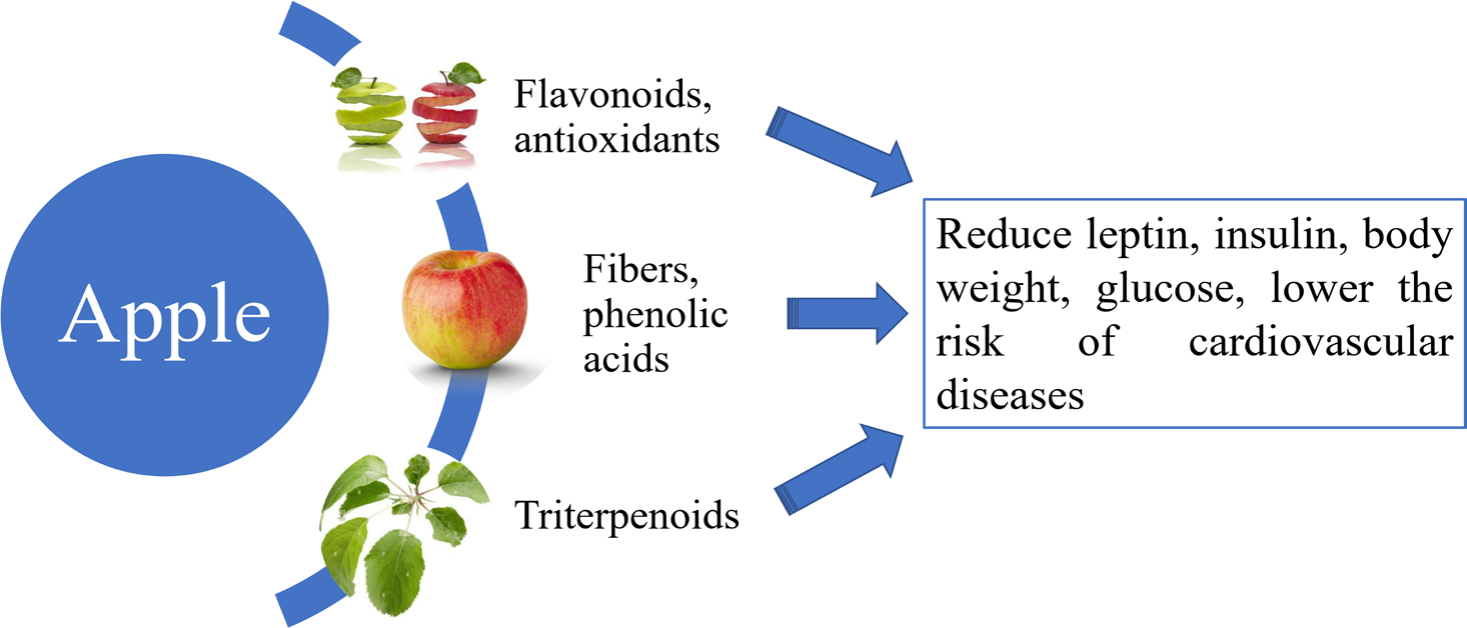
Apple peels contain flavonoids and antioxidants while apple fruit pulp contains phenolic acids and fibers, and apple leaves contain triterpenoids. These active ingredients were found to reduce leptin, insulin, body weight, blood glucose, and many of the active ingredients reduce the risk of cardiovascular diseases.

According to the old saying “an apple a day keeps the doctors away,” dietitians recommended the consumption of about 35–45 g of apples daily as that contributes to the prevention of many diseases (Shirosaki et al. 2012; Popiolek‐Kalisz et al. 2024). Obese mice fed with *M. hupehensis* leaf extract (MHLE) were found to have reduced body weight (Wu et al. 2020). Daily consumption of apple products was suggested to be effective in curbing weight gain as observed in a study on apple pomace‐fed obese rats (Cho et al. 2013). Green apple (Granny Smith) stored under low oxygen concentration reduced body weight in mice fed a high fat diet (HFD) (Soleti et al. 2020). It was found that flesh fibers can impact metabolic parameters (Figure 2) such as body mass, waist circumference, and body mass index (BMI) while polyphenol content and cyanidin were inversely associated with obesity and diabetes type 2 (Soleti et al. 2020; Josimuddin et al. 2022). As mentioned earlier, the peel has a higher antioxidant content than the flesh, therefore it was recommended to intake apple peel (whole apple) as a good natural source to improve metabolic disorders (Wu et al. 2020). In a noteworthy study, the golden delicious apple did not cause body weight loss. Moreover, the desire for food consumption was increased due to the high content of fructose which has a slower absorption rate that decreases hypothalamic satiety drive, increases food cue reactivity in the brain and the circulating level of appetite hormones (insulin, leptin and glucagon‐like polypeptide‐1) (Aksoy and Otles 2022). The fructose content of the golden delicious apple was higher than other apples; it is about 5.2 g/100 g of apple (Hermann and Bordewick‐Dell 2018).

Apple has been found to play an important and beneficial role in treating type 2 diabetes mellitus (Josimuddin et al. 2022). When tested on glucose‐loaded mice, the effect of the extract of fruit, peel, and leaf separately indicated that extracts reduced blood glucose levels, but the leaves had the highest effect. Leaves acted by suppressing glucose uptake in the intestine; at the same time, they did not show inhibitory activity against the carbohydrate digestive enzymes α‐glucosidase and α‐amylase (Shirosaki et al. 2012). Triglycerides and lipid accumulation were reduced, and fatty acid synthesis enzymes were inhibited in 3 T3‐L1 adipocytes after treatment with extracts, and there was an increase in the AMP‐activated protein kinase (AMPK) level resulting from the inhibition of liver kinase B1 and attenuation of peroxisome proliferator‐activated receptor gamma (PRAR)γ, whereas *Malus* triterpenoid reduced serum leptin, insulin, and glucose levels (Ko and Ku 2022).

In insulin‐resistant hepatoblastoma cell line (HepG2), apple reduced glucose uptake by enhancing glycogen synthesis and decreasing gluconeogenesis through the activation of IRS2/PI3K/AKT/GSK3β (the important pathway for regulating hepatic insulin‐resistance) (Li, Yang, et al. 2020). Histologically, obesity caused accumulation of lipids in the liver; therefore, the hepatocytes of obese animals were irregularly arranged in different sizes and had many fat vacuoles, increasing both hepatic steatosis and fibrosis. Granny Smith apple, apple pomace, and apple juice modified these parameters and decreased fatty acid synthase gene and stearoyl‐coenzyme A desaturase‐1 (Scd1) expressions (Cho et al. 2013; Soleti et al. 2020). Hepatocytes of obese mice given *Malus hupehensis* leaf extract (MHLE) were arranged neatly around the central vein with relatively clear morphology and few fat vacuoles (Wu et al. 2020). Apple flesh and leaf slowed fat accumulation and protected liver function by lowering serum ALT, AST, and ALP (Wu et al. 2020; Aksoy and Otles 2022). On the other hand, Aksoy and Otles showed that liver weight increased in obese rats that received green apples over those on a normal diet without apples, presumably due to glycogen accumulation in the liver. In general, there was no difference between the ratio of total lipids to liver weight when animals were given green apples or given the major flavonoids of apple (Aksoy and Otles 2022). The obese group had higher concentrations of free fatty acids, phospholipids, and triglycerides in serum than their controls fed on a normal diet. Green apples decreased the free fatty acids and phospholipids concentrations but did not change the total cholesterol level (Aksoy and Otles 2022).

On the other hand, Cho et al. showed that obese rats fed on apple pomace gained significantly less weight than obese rats without apple pomace. Apple pomace and apple juice concentrate decreased LDL and increased HDL in obese rats. Epididymal adipocyte size and subcutaneous fat pads were smaller in the obese animals fed apple pomace or juice (Cho et al. 2013). After the intervention of MHLE, the adipocytes were arranged densely; they became smaller and there was no significant difference in adipocytes morphology and size between the treated and normal diet groups (Wu et al. 2020).

Due to its high content of potassium, quercetin, and antioxidants, *Malus* can lower the risk of cardiovascular disease by reducing sodium absorption, improving vascular function, lowering serum LDL, total cholesterol, triglycerides, phospholipids, and free fatty acids, but elevating HDL and improving endothelial function (Wolfe et al. 2003; Aksoy and Otles 2022). *Malus* fibers consist of pectin and arabinoxylan, which bind and prevent the absorption of toxins in the intestine, thus affecting the gut microbiota leading to improved serum lipids (Bator et al. 2024; Popiolek‐Kalisz et al. 2024). In pilot studies, MHLE was found to improve the disturbances of blood lipids (Wu et al. 2020; Soleti et al. 2020). Granny Smith apple decreased systolic, diastolic, and mean blood pressure and reduced aortic root lesion area in HFD animals (Soleti et al. 2020). *Malus* also lowered the risk of thrombotic stroke and obstructive pulmonary diseases and subsequently reduced total mortality (Shirosaki et al. 2012; Wolfe et al. 2003).

It should be noted, however, that seed degradation catalyzed by β‐glucosidase, an enzyme naturally present in the human intestine, led to the formation of cyanide causing severe toxicity in humans (Asma et al. 2023). The above studies demonstrated that green apples reduced body weight and impacted body mass, waist circumference, and BMI. But this was not the case with golden delicious apples, which did not reduce body weight but rather increased appetite and increased levels of appetite hormones. On the other hand, many studies demonstrated that *Malus* fibers were linked to lower body weight and reduced risk of obesity. The high polyphenols content and cyanidins present in *Malus* seemed to have impacted obesity and diabetes type 2 and kept serum lipid levels under control through inhibition of fatty acid synthesis enzymes in adipocytes, thus lowering the risk of cardiovascular disease. *Malus* had anti‐oxidant properties that protected against fatty liver and decreased adipose tissue mass.

### 2.3 *Pyrus* (Pears)

*Pyrus* is commonly known as pears; its name usually reflects the geographic area such as Korean pears, Chinese pears, and Japanese pears (Hong et al. 2021). The genus *Pyrus* is known to contain 20 species and interspecific crossbreed species, but the five main species of pears are: *P. communis* in Europe, and *P. pyrifolia* , *P. bretschneideri*, *P. ussurienses*, and *P. sinkiagensis* in Asia (Nazir et al. 2020; Hong et al. 2021). European pears are characterized by a stretched, full‐bodied structure with soft and smooth flesh, few stone cells, and a stronger aroma and flavor, while Asian pears have circular, crisp flesh, with a sandy surface, high sugar content, low acid content, minimal aroma, and mild flavor (Nazir et al. 2020; Hong et al. 2021).

#### 2.3.1 Effect on Obesity and Metabolic Disorder

Pears were found to reduce body weight in obese rats, and there was a reduction in food intake; thus, it was suggested that too much consumption of pears made people thin and weak and brought out diarrhea (Hong et al. 2021). The chlorogenic content of fermented and unfermented *P. ussuriensis* prevented extra body weight gain in obese mice, possibly due to the reduction of total gastric inhibitory peptides (GIP), gamma‐glutamyl transferase, insulin, TNFα, IL‐6, leptin, and the secretion of ghrelin and expression of lipogenesis genes. Peng et al. showed that pear fruit pomace caused an increase in the abundance of gut microbiota (such as *Akkermansia*, *Bacteroides*, *Parabacteroides*, *Alistipes* and *Alloprevotella*) which is considered an important internal environmental factor to prevent the occurrence of obesity (Boby et al. 2022; Peng et al. 2022).

*P. bretschneideri, P. ussuriensis* peel had a higher effect in reducing blood glucose than pulp due to the more potent inhibitory effect of α‐glucosidase, which delays the digestion and absorption of carbohydrates. Similarly, *P. communis* , *P. pyrifolia* juice had a similar impact (Wang et al. 2015; Rutkowska and Olszewska 2023). Boby et al. showed that the hypoglycemic effect of both fermented and non‐fermented *P. ussuriensis* was attributed to enhanced GLUT4 mRNA gene expression, thus preventing insulin resistance and diabetes type 2 (Boby et al. 2022). A diabetic group of mice showed glucose intolerance with a high increase in blood glucose level that continued for 3 h. *P. communis* fruit was effective in depressing the peak value of blood sugar at 90 min while *P. ussuriensis* peel improved glucose tolerance at 60 min. These results were ascribed to an increase in the secretion of insulin (Velmurugan and Bhargava 2013; Wang et al. 2015).

Much research showed that a streptozotocin‐induced type 2 diabetes or high‐fat diet elevated serum LDL, VLDL, TC, and TG and decreased HDL levels in animal models. *P. ussuriensis* pomace or fermented fruit and *P. communis* fruit, peel, and pulp reduced these parameters but elevated HDL. Wang et al. showed that peels had a higher effect in adjusting these parameters than pulp (Boby et al. 2022; Peng et al. 2022; Velmurugan and Bhargava 2013; Wang et al. 2015). LDL, TG, and TC reduction were interpreted in many ways. For example, these parameters can be reduced by a reduction in the absorption of glucose and cholesterol into the blood. Velmurugan and Bhargava (2013) found that *Pyrus* contained several phenolic and flavonoid compounds with anti‐diabetic and hypolipidemic properties, while Wang et al. concluded that the adjustment in serum superoxide dismutase (SOD) and thiobarbituric acid‐reactive substance (TBARS) reduced the risks of diabetes complications (Velmurugan and Bhargava 2013; Wang et al. 2015).

Obesity causes abnormal fat accumulation and affects the organization of fat cells, which become disordered and assume different sizes, while *P. ussuriensis* treatment significantly reduced the size of fat cells in a dose‐dependent manner by lowering TG storage in adipocytes (Boby et al. 2022; Peng et al. 2022).

Hyperlipidemia is often associated with obesity and hyperglycemia, and this combination increases the risk factor for cardiovascular disease (Wang et al. 2015). *P. pyrifolia* fruit had an antihypertensive effect by binding to the active site of angiotensin‐converting enzyme (ACE) (Mahdy et al. 2023). Pear helped in curing cardiovascular disorders by reducing high blood pressure and stroke (Nazir et al. 2020). *P. ussuriensis* treated groups had a low value of atherogenic index (AI) and this was indicative of pear lipid‐lowering properties associated with cardioprotective potential (Boby et al. 2022). *Pyrus* pomace reduced lipid accumulation in 3T3‐adipocyte cell line (Hong et al. 2021). The weight of the liver was reduced in the *P. ussuriensis* treated versus non‐treated HFD‐fed rats (Hong et al. 2021; Boby et al. 2022). The histopathological alterations were detected in obese mice, but after administration of *P. ussuriensis* extract, the liver was arranged regularly with hepatic cords and clear boundaries between cells (Boby et al. 2022; Peng et al. 2022). Further studies on other pear species, like *P. bretschneider* and *P. sinciagensis*, are needed to help discover new medicinal benefits of pears.

The findings from the above studies indicated that *Pyrus* not only prevented weight gain but also reduced weight mostly due to reducing food intake. Combined with an effect on gut microbiota, it prevented obesity. Moreover, *Pyrus* seemed to reduce blood glucose, prevent insulin resistance, and diabetes type 2. On the lipid profile, *Pyrus* reduced LDL‐C, increased HDL‐C, and reduced the size of fat cells.

### 2.4 *Eriobotrya* (Loquat)

*Eriobotrya* plants originated in both southwestern China and southeastern Asia during the previous geological age, then migrated to the Middle East, Europe, and America (Lin et al. 2010; Idrees et al. 2021). There are 24 species of loquat, the main species of which are *E. japonica*, *E. cavaleriei* , *E. bengalensis* , *E. stipularis*, *E. dubia* , *E. malipoensis, and E. daduheensis* (Idrees et al. 2021).

#### 2.4.1 Effect on Obesity and Metabolic Disorder

The extract of *E. japonica* fruit, leaf, and seed effectively reduced body weight in obese rats, presumably due to the higher content of flavonoids that may induce satiety and lower food intake (Atkins and Wannamathee 2020). Serum glucose was also reduced in obese rats after applying fruit, seed, or leaf extract separately, due to the high content of fibers, total flavonoids, and total phenols, which likely inhibited α‐glucosidase (Shih et al. 2010; Tanaka et al. 2008). Li, Li, et al. (2020) showed that triterpenoids extracted from the leaves had a hypoglycemic effect by increasing the transcription of both PPAR‐γ and GLUT2 and inhibiting intestinal sugar digestion, whereas Abdelrahman et al. (2023) found that the fruit had a better hypoglycemic effect than leaves and seeds (Abdelrahman et al. 2023; Li, Li, et al. 2020).

Triterpenoids extracted from *E. japonica* fruit ameliorated the impaired glucose tolerance after 30 min and insulin sensitivity in obese mice. For example, Tanaka, et al. confirmed that the seed had a hypoglycemic effect; it suppressed blood glucose levels and improved glucose tolerance in the type 2 diabetes KK‐A^y^ mice model by delaying glucose absorption in the intestines and improving insulin resistance (Shih et al. 2010; Tanaka et al. 2008).

**FIGURE 3:**
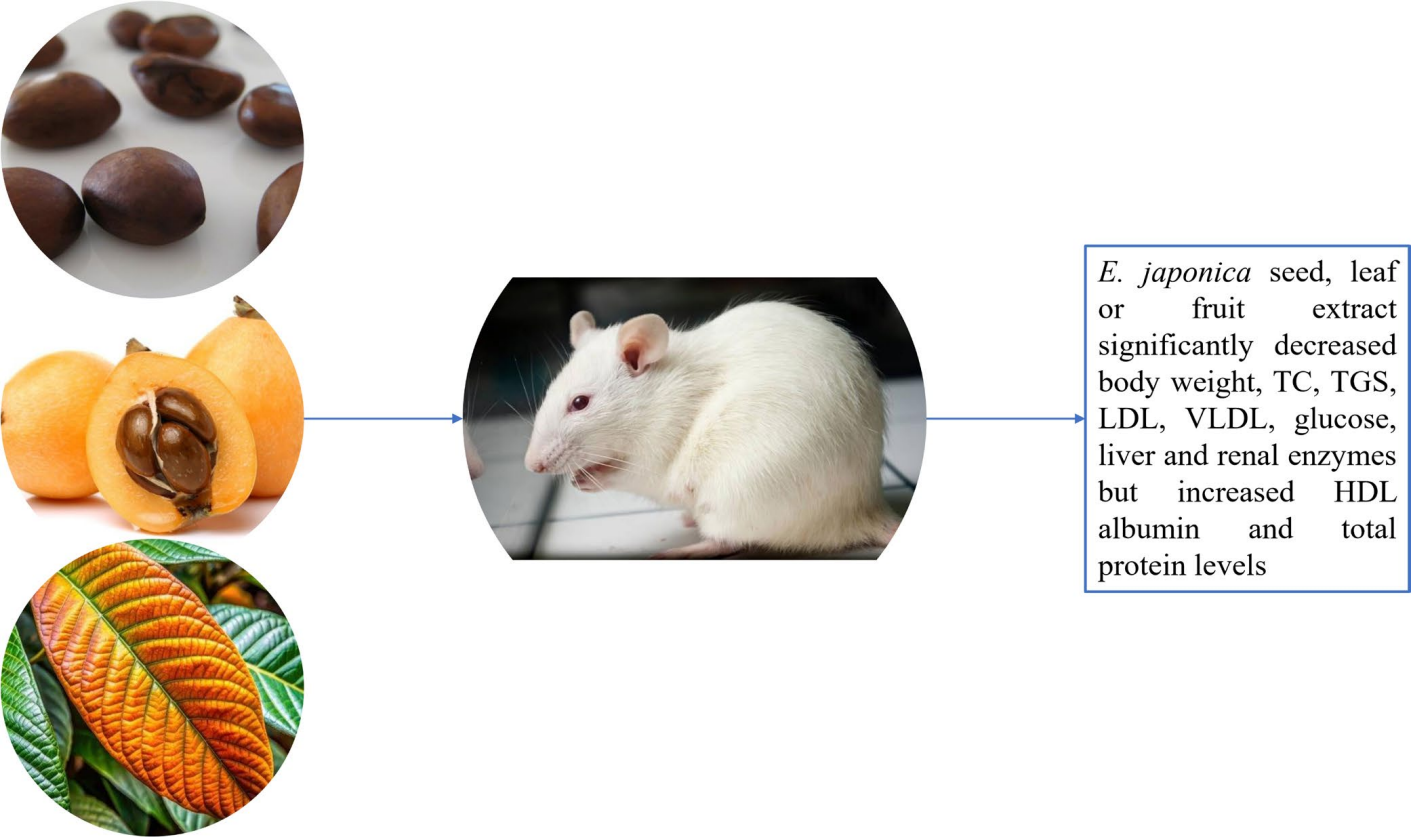
Ethanol extract of loquat fruits, seeds, and leaves was found to decrease body weight, decrease total cholesterol (TC), triglycerides (TG), low density lipoprotein (LDL) and very low density lipoprotein (VLDL), serum glucose, liver and renal enzymes, but increased high density lipoprotein (HDL), albumin and total proteins of rats.

The extract of *E. japonica* seeds, leaves, and fruits reduced TC, LDL, VLDL, and TG and elevated HDL (Abdelrahman et al. 2023; Tanaka et al. 2008). Abdelrahman et al. found that the fruit caused a better reduction in TC and LDL, while seeds and leaves caused a better reduction in serum triglyceride (Figure 3). On the other hand, Li et al. showed that triterpenoids extracted from the leaves increased the transcription of PPAR‐γ which regulated lipid metabolism (Abdelrahman et al. 2023; Li, Li, et al. 2020).

*E. japonica* extracts suppressed the increase in liver enzymes such as GOT, ALT, ALP, and GGT. The increase in these enzymes is a symptom of non‐alcoholic fatty liver disease, which is considered one of the obesity‐associated complications (Abdelrahman et al. 2023; Elsherif et al. 2023). *E. japonica* extract significantly suppressed lipid and triglyceride accumulation in the liver and decreased adipocyte hypertrophy by decreasing the concentration of leptin and increasing the transcription of PPAR‐γ (Shih et al. 2010). Leaf and fruit extracts significantly reduced the weight of many organs, such as the liver, spleen, and lungs, which showed an increase when rats were fed on HFD. Histologically, obese rats had hepatic vacuolar degeneration and sinusoidal leukocytes, which were ameliorated by the extract of *E. japonica* leaf and fruit (Elsherif et al. 2023).

Albuminuria, proteinuria, increased serum creatinine, uric acids, and urea are parameters associated with obesity. Administration of *E. japonica* seed, leaf, or fruit extract to obese rats also significantly ameliorated the perturbed renal markers (Abdelrahman et al. 2023; Elsherif et al. 2023).

The administration of *E. japonica* leaf extract alleviated the cardiotoxicity markers when cardiotoxicity was induced by CCL4 in rats; it reduced the elevation in lactate dehydrogenase (LDH), creatine kinase (CK) and malondialdehyde (MDA). Histologically, many areas of necrotic muscle fibers and clogged blood arteries were detected, but the leaf extract reduced these changes and kept the normal histology of heart muscle in rats. Elsherif et al. demonstrated that the weight of the heart decreased in obese rats after applying leaves and fruit extract (Elsherif et al. 2023; Shahat et al. 2023).

Just like *Pyrus*, *Eriobotria* reduced body weight; it seemed to reduce food intake or stimulate the satiety center in the hypothalamus. Extracts of fruit, seed, or leaf all reduced serum glucose, ameliorated glucose tolerance, and inhibited α‐glucosidase. On lipids, *Eriobotria* reduced LDL and many lipid profile parameters but increased HDL and increased the transcription of PPAR‐γ that regulated lipid metabolism.

### 2.5 *Crataegus* (Hawthorn)

The genus *Crataegus*, including more than 1000 species worldwide, has been identified and confirmed in China with red fruit and some yellow individuals. The main species are *C. cuneata, C. dachurica, C. maximowiczii*, and *C. monogyna* , but the most widely distributed species is *C. pinnatifida* (Guo and Jiao 1995).

#### 2.5.1 Effect on Obesity and Metabolic Disorder

In a pilot study, a high‐fat diet caused an increase in rat weight and increased their food intake. When treated with a mixture of leaves of *Crataegus pinnatifida* and *Laurus nobilis* , they showed reduced body weight, reduced food intake, and reduced insulin secretion. *C. monogyna* fruits also caused similar effects (Ibrahim et al. 2022; Feng et al. 2022). Streptozotocin (STZ)‐treated mice lost weight, but hawthorn seeds increased the diabetic mice's weight by increasing ghrelin and decreasing motilin and gastrin (Wahabi et al. 2023). Moreover, in streptozotocin‐treated or HFD‐treated animal models, hawthorn fruits and seeds reduced blood glucose (Feng et al. 2022; Wahabi et al. 2023). Furthermore, *C. monogyna* leaves and flowers and *C. laevigata* fruits had a hypoglycemic effect by enhancing glucose tolerance, significantly reducing postprandial hyperglycemia at 60 min by blocking intestinal glucose absorption and inhibiting pancreatic α‐amylase secretion (Radi et al. 2023; Alaghawani and Naser 2013).

*C. pinnatifida* and *C. monogyna* fruits administration reduced serum TC, LDL, and TG and increased HDL in obese rats fed on HFD (Radi et al. 2023), and the mixture of leaves of *C. pinnatifida* and *L. nobilis* showed similar results (Ibrahim et al. 2022). Feng et al. reported that oleic acid of the fruits alleviated serum lipid disturbance (Feng et al. 2022). *C. pinnatifida* fruits also reduced the accumulation of lipid droplets in hepatocellular carcinoma cell lines (HepG2); therefore, it was suggested that hawthorn fruit has a protective effect on HepG2 cells due to their content of antioxidants (Feng et al. 2022). *C. monogyna* administration of fruits and leaves extracts separately reduced triglycerides, cholesterol, and LDL concentrations and raised HDL levels, indicating that *C. monogyna* has hypocholesterolemic and vasoprotective activities (Remita et al. 2021). It was also shown that *C. monogyna* fruits and leaves reduced hypercholesterolemia and hyperlipidemia, which are the risk factors for atherosclerosis and damage of the endothelial cells, and reduced the atherogenic index (Ibrahim et al. 2022; Feng et al. 2022).

Hawthorn maintained cardiac hemostasis because it has blood‐thinning properties. For example, *C. monogyna* leaves and fruits increased the heart antithrombin III and decreased the levels of serum sPECAM‐1, an endothelial sclerosis indicator, and these effects were attributed to their high contents of flavonoids and phenolic compounds. This blood thinning effect was substantiated by the finding that another species, *C. oxyacantha* , had effective antiplatelet activity and reduced levels of thromboxane B2 in the serum (Wang et al. 2013). Similar to *C. pinnatifida* , *C. monogyna* fruit extract was found to possess therapeutic potential against rat myoblast cells due to its antioxidant contents (Ravikumar et al. 2022; Rababa'h et al. 2020).

In obese rats that have elevation in serum AST, ALT, GGT, ALP, and bilirubin, *C. monogyna* fruits significantly alleviated the disturbance in these parameters (Ibrahim et al. 2022; Wahabi et al. 2023). In obese rats, the weight of the liver increased significantly as compared with normal rats, but *C. monogyna* consumption reduced the liver weight in these animals, and a mixture extract of leaves and fruits had a potential effect in reducing liver weight as compared to those of normal rats (Wahabi et al. 2023; Rababa'h et al. 2020). Histologically, hyperlipidemic rats exhibited severe changes in liver structure, such as hepatocyte necrosis, cytoplasmic vacuolization, cell degeneration, and loss of cell boundaries. Moreover, a large accumulation of fat in the hepatocytes was observed in the form of droplets, whereas, in the *C. monogyna* and *C. pinnatifida* fruit‐treated group, the fat droplet particles were smaller, less numerous, and the cells were arranged more regularly due to reduced fat accumulation (Feng et al. 2022; Wahabi et al. 2023). Also, the weight of the kidney in obese rats increased significantly as compared with that of normal rats, and there was an increase in their serum uric acids and urea, but reduced creatinine. The consumption of *C. monogyna* fruits reduced the weight of the kidney and alleviated the disturbance in these parameters (Feng et al. 2022).

The above studies demonstrated that *Crataegus* fruits reduced the body weight of obese animals and decreased food intake and insulin secretion. They also reduced blood glucose and enhanced glucose tolerance mostly because they blocked glucose absorption and inhibited pancreatic α‐amylase. On the lipid profile, all parameters were reduced except for HDL‐C, which was increased, and the liver weight in obese rats was reduced, and lipid accumulation in HepG2 cells was also reduced.

### 2.6 *Cydonia* (Quince)

*Cydonia oblonga* (quince), also called kinashi, soil papaya, biye, bahi (Urdu) and safarjal (Arabic), is a seasonal fruit tree, cultivated in gardens under warm temperatures (Aslam and Hussain 2013; Abliz et al. 2014). The tree fruit looks like pears. Mature quince has a pleasant, durable, and powerful flavor (Amerizadeh et al. 2022; Ashrafi et al. 2013). It is believed that quince originated in northern Iran, Caucasus, Armenia, Azerbaijan, Turkmenistan, southwest Russia, and west of Anatolia and Greece, but it also grew in the sub‐Himalayan regions of south Asia (Ashraf et al. 2019; Mohebbi et al. 2019). The fruit is consumed fresh but can be consumed when cooked or processed as jam or jelly (Silva et al. 2002).

#### 2.6.1 Effect on Obesity and Metabolic Disorder

The body weight, food intake, and body fat mass percentage in a high‐fat diet group of animals were significantly higher than those in a normal diet group. Treatment with *Cydonia oblonga* fruit significantly reduced body weight, food consumption, and decreased body fat mass percentage by 11.3% compared to the untreated obese diet group (Lee et al. 2022). Lee et al. showed that chlorogenic acid was the main component in *C. oblonga* fruit that exhibited anti‐obesity effects (Lee et al. 2022). *C. oblonga* seed or leaves extract caused no effect on reducing body weight, food, and water consumption compared to the untreated group (Abliz et al. 2014; Mohebbi et al. 2019). On the other hand, Lee et al. (2022) showed that treatment with quince fruit decreased body weight, total white adipose tissue, and fat tissue in obese mice by lowering serum leptin (hunger hormone) (Lee et al. 2022).

*C. oblonga* fruit extract showed antidiabetic activity as evidenced by its capacity to inhibit the α‐glucosidase (Sakhri et al. 2021). Abed, et al. interpreted the anti‐diabetic activity of quince bark against α‐glucosidase and α‐amylase as attributed to the fact that they are rich sources of phenolic compounds and flavonoids along with other bioactive compounds, while Amerizadeh, et al. reported that quince fruits and seeds improved glucose metabolism by stimulating the PI3K/AKT signaling pathway (Amerizadeh et al. 2022; Abed et al. 2022). The elevated serum glucose in diabetic and obese animals was reduced by *C. oblonga* fruits. Lee, et al. observed that the hypoglycemic effect of quince fruit was associated with reduced serum insulin and leptin, increased adiponectin, and increased AMPK activation (Mohebbi et al. 2019; Lee et al. 2023).

Both streptozotocin injection and feeding a high‐fat diet led to the development of hyperlipidemia exhibited as increased TG, TC, LDL‐C, and decreased HDL‐C when compared to the normal animal group. *C. oblonga* fruit decreased serum TG, TC, and LDL‐C, but significantly increased HDL‐C levels, and *C. oblonga* leaves had a similar antihyperlipidemic effect (Mirmohammadlu et al. 2015; Abliz et al. 2014). Lee et al. showed that quince fruit reduced TG and elevated HDL but did not lower TC and LDL, whereas Amerizadeh et al. showed that total flavonoids of quince fruit and leaves could adjust the lipid profile in hyperlipidemic rats (Amerizadeh et al. 2022; Lee et al. 2022).

ALT, AST, and ALP decreased in rats treated with quince fruit (Sakhri et al. 2021; Adiban et al. 2019). The serum total protein level was lower in the hyperlipidemic mice group than in normal mice, but quince leaf treatment increased the level of total protein (Abliz et al. 2014). Histologically, the fatty liver was white‐pinkish, had a tense capsule with swelling tissue, was less flexible than the normal liver, and had a greasy feeling, with many fat vacuoles, increased liver volume, and some cell nuclei apparently with fatty degeneration (Abliz et al. 2014). Quince restored liver volume to normal levels, liver cell cords arranged normally, and the overall cell degeneration had significantly improved (Amerizadeh et al. 2022; Sakhri et al. 2021).

The levels of serum urea, uric acid, and creatinine as markers of renal dysfunction were elevated in the diabetic rats, but quince fruit extract significantly inhibited the increase in kidney function markers (Mirmohammadlu et al. 2015; Sakhri et al. 2021).

In hypercholesterolemic rabbits, tunica albuginea showed thickening and an increase in intertubular connective tissue as significant structural changes in testicular sections. Many other changes, including disorganization of germinal epithelium, abundant spermatogonia and primary spermatocytes along the germinal epithelium, reduced thickness of the germinal epithelium, and vasodilatation of vessels in the interstitial tissue, were also observed. Quince leaf protected rabbit testes and spermatogenesis from damage caused by hypercholesterolemia (Ashrafi et al. 2013). Also, quince leaves and seeds have an antihypertensive effect by ameliorating high blood pressure (Amerizadeh et al. 2022).

*Cydonia* fruits seem to decrease body weight, food consumption, and fat mass in experimental animals. This antiobesity effect has been attributed to the reduction of the hunger hormone leptin, presumably resulting from the presence of flavonoids and phenolic acids such as chlorogenic acid in the fruit. *Cydonia* fruits also have antidiabetic activity that resulted from the inhibition of α‐glucosidase, increased adiponectin, and activation of the AMPK pathway.

In summary, Table 2 lists, for the benefit of the reader, the major phytochemicals in the different species mentioned in this review and the reported biological effects in terms of obesity and metabolic disorders.

**TABLE 2:** Summary of the major phytochemicals and the biological effects of Maloideae species and varieties.

Maloideae genera	Species/Part	Major phytochemicals	Reported biological effects
*Malus* (Apple)	*M. domestica* (fruit: peel, flesh, pomace)	*Leaf* Phlorizin, phloretin, flavonoids (Shirosaki et al. 2012) *Pomace* Chlorogenic acid, catechin/epicatechin, quercetin glycosides, procyanidins (Cho et al. 2013) *Fruit* Quercetin, kaempferol, myricetin (Aksoy and Otles 2022)	Leaf reduced blood glucose levels through inhibition of glucose absorption (Shirosaki et al. 2012) Pomace reduced body weight and body fat mass and improved serum lipid profile (Cho et al. 2013) Fruit reduced obesity and fat accumulation, improved serum lipid profile (Aksoy and Otles 2022)
	*M. hupehensis* leaf	Phlorizin, phloretin, quercetin and its glycosides (flavonols), chlorogenic acid (phenolic acid) (Wu et al. 2020)	Leaf reduced body weight gain and fat accumulation, improved serum lipid profile and improved hepatic lipid metabolism (Wu et al. 2020)
	Granny smith apple flesh fibers	Polyphenols, flavonoids, particularly: quercetin glycosides, phloridzin, procyanidins, chlorogenic acid, catechin/epicatechin (Soleti et al. 2020)	Fruit decreased metabolic parameters such as body mass, waist circumference, and body mass index, reduced systolic, diastolic, and mean blood pressure and reduced the extent of atherosclerotic lesions in the aortic root (Soleti et al. 2020)
	Fuji apple peel	Flavonoids, dihydrochalcones like phloridzin, triterpenoid (Bator et al. 2024)	Peel caused anti‐obesity effects by reducing fat accumulation (Ko and Ku 2022), improved vascular health and promoted beneficial bacteria (Bator et al. 2024)
*Pyrus* (Pears)	*P. ussuriensis* fruit	*Fruit* Phenolic compounds (chlorogenic acid, arbutin, rutin, and catechins), flavonoids and organic acids and antioxidants (Boby et al. 2022) Fermentation increased the bioavailability and concentration of some of these active phenolics (Boby et al. 2022) *Pomace* Cellulose, hemicellulose, and lignin (Peng et al. 2022), phenolics (e.g., chlorogenic acid, catechins, quercetin derivatives) (Peng et al. 2022)	Fermented fruit extract significantly reduced body weight gain, fat accumulation, and adipocyte hypertrophy in high‐fat diet (HFD)‐induced obese rats, improved serum lipid profile, enhanced insulin sensitivity and reduced fasting glucose levels and increased abundance of beneficial bacteria (Boby et al. 2022) Pomace alleviated hepatic steatosis and reduced adipocyte hypertrophy, enhanced glucose tolerance and insulin sensitivity (Peng et al. 2022)
	*P. communis*	*Fruit* Phenolic compounds (chlorogenic acid, arbutin, catechins, flavonoids like quercetin derivatives), Glycosides and tannins (Nazir et al. 2020; Velmurugan and Bhargava 2013) Flavonoids, alongside triterpenes, β‐carotene, lutein, and zeaxanthin (Nazir et al. 2020)	Fruit had anti‐diabetic effect, hypolipidemic effect and prevented excessive weight loss (Velmurugan and Bhargava 2013) Reduced risk of chronic diseases such as cardiovascular disease and diabetes (Nazir et al. 2020)
	*P. pyrifolia*	*Fruit* Polyphenols, flavonols, dihydrochalcones, proanthocyanidins, anthocyanins and phenolic acids (Rutkowska and Olszewska 2023) Rutin, isoquercitrin, isorhamnetin‐3‐O‐β‐D‐glucoside, chlorogenic acid, quercetin, cinnamic acid (Mahdy et al. 2023)	Fruit modulated insulin signaling (Rutkowska and Olszewska 2023) Fruit extract inhibited key enzymes involved in metabolic syndrome, including: α‐glucosidase, α‐amylase, pancreatic lipase (Rutkowska and Olszewska 2023), and blood pressure regulating enzyme such as: angiotensin‐converting enzyme (ACE), and renin (Mahdy et al. 2023)
*Eriobotrya* (Loquat)	*E. japonica*	*Seed* Polyphenolic compounds, including: chlorogenic acid, cyanidin glycosides, epicatechin, epigallocatechin gallate, and procyanidin B2 (Abdelrahman et al. 2023), amygdalin (Tanaka et al. 2008) *Frui*t Rich in sugars, organic acids, carotenoids, flavonoids, phenolic acids, and vitamins (Abdelrahman et al. 2023) *Leaf* Rich in phenolics and triterpenes (Shih et al. 2010; Li, Li, et al. 2020) Kaempferol‐3‐O‐rhamnoside, quercetin‐3‐O‐rhamnoside, quercetin‐3,7‐di‐O‐glycerides and roseoside (Shahat et al. 2023)	Seed reduced body weight gain, serum glucose, and lipid markers (TC, TGs, LDL, VLDL), improved HDL, albumin, and total protein levels, lowered liver and kidney enzymes level (Abdelrahman et al. 2023) Seed improved glucose tolerance (Tanaka et al. 2008) Fruit lowered kidney markers such as urea, creatinine, and uric acid, and liver enzyme like AST, ALT, and ALP in hyperlipidemic rats (Abdelrahman et al. 2023; Elsherif et al. 2023) Fruit ameliorated serum glucose level and lipid profile (Abdelrahman et al. 2023; Elsherif et al. 2023) Fruit prevented histopathological changes (Elsherif et al. 2023) Leaf improved insulin resistance, ameliorated hyperglycemia and reduced hyperlipidemia (Abdelrahman et al. 2023; Shih et al. 2010) Leaf reduced adipose tissue mass, upregulated PPARs and GLUT2, and improved OGTT results and insulin tolerance test (Shih et al. 2010; Li, Li, et al. 2020) Leaf markedly reduced CCl_4_‐induced cardiac tissue damage, lowered cardiac injury markers‐lactate dehydrogenase (LDH) and creatine kinase (Shahat et al. 2023)
*Crataegus* (Hawthorn)	*C. monogyna*	*Leaf* Flavonoids (e.g., rutin, quercetin derivatives), phenolic acids (e.g., chlorogenic acid, gallic acid) and tannins, saponins, terpenoids and glycosides (Radi et al. 2023; Remita et al. 2021) *Fruit* Rich in phenolic compounds and flavonoids, chlorogenic acid, caffeic acid, rutin, quercetin derivatives, also contains procyanidins and organic acids (Wahabi et al. 2023; Remita et al. 2021)	Leaf reduced blood glucose levels, improved body weight, enhanced glucose tolerance and improved liver and kidney biochemical parameters (Radi et al. 2023; Remita et al. 2021) Fruit extract exerted anti‐obesity and improved serum lipid profile (Wahabi et al. 2023) Fruit ameliorated histological changes in liver tissue and reduced the weight of the kidney in obese animals (Feng et al. 2022; Wahabi et al. 2023) Fruit improved biochemical markers related to liver/kidney function (Wahabi et al. 2023; Remita et al. 2021) Fruit had therapeutic potential in cardiovascular disease prevention/treatment (Wang et al. 2013; Ravikumar et al. 2022)
	*C. pinnatifida*	*Leaf* Flavonoids (e.g., hyperoside, vitexin, rutin, quercetin derivatives), phenolic acids (chlorogenic acid, gallic acid), triterpenes and procyanidins (Ibrahim et al. 2022) *Fruit* Contains phenolic compounds and citric acid, malic acid, succinic acid and tartaric acid (Feng et al. 2022)	Leaf extract reduced body weight gain, improved lipid profile, reduced blood glucose levels, improved liver function markers and reduced steatosis in liver tissue (Ibrahim et al. 2022) Fruits lowered serum lipid, increased HDL and improved histopathological features of liver tissue (Feng et al. 2022)
	*C. laevigata*	*Leaf* Flavonoids (hyperoside, rutin, quercetin, vitexin derivatives), phenolic acids (chlorogenic acid, caffeic acid, gallic acid), procyanidins, triterpenes and saponins (Alaghawani and Naser 2013)	Leaf reduced fasting blood glucose levels, improved glucose tolerance, restored the body weight loss caused by diabetes (Alaghawani and Naser 2013)
*Cydonia* (Quince)	*C. oblonga*	*Leaf* Flavonoids (quercetin, kaempferol derivatives), phenolic acids (chlorogenic acid, caffeic acid, gallic acid), tannins and saponins (Abliz et al. 2014) *Fruit* Flavonoids (rutin, quercetin, kaempferol derivatives), phenolic acids (caffeic acid, chlorogenic acid, gallic acid) and tannins (Mohebbi et al. 2019; Lee et al. 2022) *Bark* Phenolic compounds (chlorogenic acid, gallic acid, caffeic acid), flavonoids (quercetin, rutin), tannins, saponins, alkaloids, glycosides, steroids and terpenoids (Abed et al. 2022)	Leaf extract shows hypolipidemic and liver‐protective effects (Abliz et al. 2014) Fruit reduced body weight gain and adipose tissue mass, reduced fasting blood glucose in diabetic rats, improved glucose tolerance, also demonstrated beneficial trends in lipid regulation (Mohebbi et al. 2019; Lee et al. 2022) Fruit suppressed adipogenesis, reduced lipid accumulation, enhanced lipid catabolism through AMPK pathway activation (Lee et al. 2023) Fruit improved liver function, decreased ALT, AST and ALP and protected hepatocyte integrity (Sakhri et al. 2021; Adiban et al. 2019) Fruit inhibited the increase in kidney function markers (Mirmohammadlu et al. 2015) Bark inhibited both α‐amylase and α‐glucosidase (Abed et al. 2022)

## 3 Conclusions

Maloideae, the apple subfamily, genera are associated with good human health. The in vitro and in vivo experiments suggested that they play a positive role in disease prevention and in the treatment of obesity, obesity complications, and to some degree, the metabolic disorders. This review recapitulated previous studies and focused on studies using animal models and cell lines. Apple, one of the most consumed fruits in the world, is the source of functional ingredients, such as fibers, minerals, vitamins, and phenolics. The consumption of whole apples may promote weight loss and lower the chance of getting many chronic conditions, including diabetes, heart disease, and fatty liver diseases. Pears, an old and new fruit, have phytochemicals that show beneficial effects on various diseases and have a strong potential against hyperglycemia, hypercholesterolemia, and hypertension by affecting responsible factors such as inhibiting α‐glucosidase, enhancing GLUT4 mRNA gene expression, and inhibiting ACE and adjusting serum SOD and TBARS. A range of bioactivities has been reported for different parts of loquat, since many important compounds have been isolated from this plant including ursolic acid, chlorogenic acid, quercetin glycoside, and its derivatives. Irrespective of the source used, loquat fruit, leaves, or its processed food products may have pharmacological activities such as antioxidant, hypolipidemic, anti‐diabetic, and alleviates the cardiotoxicity markers.

This review also shows that *Crataegus and Cydonia* are types of functional foods that have an established position in human nutrition, which was confirmed by scientific studies. The fruits or leaves of these plants have an antihyperglycemic effect attributed to inhibiting both α‐glucosidase and α‐ amylase, and their effect against obesity was evident and supported by scientific findings. The beneficial effect of *Crataegus* on the heart due to increasing the heart antithrombin III and decreasing the levels of serum sPECAM‐1 was documented. The beneficial effects of Maloideae plants against hyperlipidemia, hypercholesteremia, and liver enzyme elevation were reported by many studies. Current research findings also suggest potential benefits of these nutritional plants with respect to weight management, blood sugar regulation, and lipid profile improvement. The overall evidence remains somewhat limited; therefore, further studies including clinical cases and laboratory experiments are required to better understand the full potential of these plants on obesity and obesity‐related diseases. The role of the fruits of these plants should not be looked upon as an alternative to the available approved therapy for obesity and metabolic disorder but rather as adjunct therapy that may reduce the cost to the healthcare system and to individual patients and reduce the side effects of marketed drugs.

## Author Contributions

**Zainab R. Abdelrahman:** conceptualization (equal), data curation (equal), formal analysis (equal), investigation (equal), writing – original draft (equal). **Mai S. Maaliah:** data curation (equal), formal analysis (equal), investigation (equal), writing – original draft (equal). **Shtaywy S. Abdalla:** methodology (equal), project administration (equal), supervision (equal), validation (equal), visualization (equal), writing – review and editing (equal).

## Data Availability

Data available on request from the authors.
